# The comprehension and production of quantifiers in isiXhosa-speaking Grade 1 learners

**DOI:** 10.4102/sajcd.v63i2.138

**Published:** 2016-05-20

**Authors:** Joanine Nel, Frenette Southwood

**Affiliations:** 1 Department of General Linguistics, Stellenbosch University, South Africa

## Abstract

**Background:**

Quantifiers form part of the discourse-internal linguistic devices that children need to access and produce narratives and other classroom discourse. Little is known about the development - especially the prodiction - of quantifiers in child language, specifically in speakers of an African language.

**Objectives:**

The study aimed to ascertain how well Grade 1 isiXhosa first language (L1) learners perform at the beginning and at the end of Grade 1 on quantifier comprehension and production tasks.

**Method:**

Two low socioeconomic groups of L1 isiXhosa learners with either isiXhosa or English as language of learning and teaching (LOLT) were tested in February and November of their Grade 1 year with tasks targeting several quantifiers.

**Results:**

The isiXhosa LOLT group comprehended *no/none, any* and *all* fully either in February or then in November of Grade 1, and they produced all assessed quantifiers in February of Grade 1. For the English LOLT group, neither the comprehension nor the production of quantifiers was mastered by the end of Grade 1, although there was a significant increase in both their comprehension and production scores.

**Conclusion:**

The English LOLT group made significant progress in comprehension and production of quantifiers, but still performed worse than peers who had their L1 as LOLT. Generally, children with no or very little prior knowledge of the LOLT need either, (1) more deliberate exposure to quantifier-rich language or, (2) longer exposure to general classroom language before quantifiers can be expected to be mastered sufficiently to allow access to quantifier-related curriculum content.

## Introduction

Although language acquisition is fast and efficient until the age of approximately five years, becoming a proficient speaker of one’s mother tongue is an extended process which continues up to approximately the age of nine years (Berman, 2004). Later-developing language skills include the ability to use low-frequency syntactic structures such as passive constructions, subordinate clauses, and low-frequency adverbial conjunctions; past perfect marking and modal auxiliaries (Nippold, 2004); and noun phrase elaboration (Khorounjaia & Tolchinsky, 2004). Quantifiers, a further later-developing syntactic category, form part of the discourse-internal linguistic devices children need to (1) contrast and differentiate characters and objects within narratives and other spoken and written texts and (2) describe quantities in mathematical literacy. Little is known about the development of quantifiers in child language, especially the production thereof, and particularly in children who speak an African language. This study investigates the comprehension and production of quantifiers by young school-going speakers with isiXhosa as mother tongue.

Young children in South Africa generally have low literacy levels (Department of Basic Education, 2014; Olivier, 2009), which are linked to poor language skills (Klop & Tuomi, 2007), specifically to an inability to comprehend and produce some of the above mentioned later-developing constructions used by the children’s teachers in the classroom. Upon entering school, children are exposed to classroom discourse, which (1) comprises more formal spoken discourse and writing, (2) is often decontextualised in nature (see Naremore, Densmore & Harman, 1995), and (3) contains complex syntax (see Dunn Davison *et al*. 2012). Children acquire greater syntactic proficiency if they are exposed to linguistic input frequently containing multiclausal utterances rather than to simplified speech (Huttenlocher, Vasilyeva, Cymerman & Levine, 2002). There is thus an ‘on-going, cyclical relationship between literacy and later language development …, a process that is heavily supported by … [*language input*]’ (Nippold, 2004, p. 6).

Apart from linguistic input, language and literacy skills are also affected by socioeconomic status (SES). Children growing up in low SES environments may be poverty-situated in terms of not only their physical conditions but also their development of language and literacy skills (Aram & Biron, 2004; Farran, 1982; Klop & Tuomi, 2007). Tough (1982) states that ‘children from disadvantaged sections of the community [*do not*] generally lack language but their expectations about using language do not support learning’ (p. 13). The language and socialisation style to which children are exposed, (1) influence the type of language that the child will finally master, and (2) propagate certain information-processing strategies that affect later learning (Farran, 1982). Children from low SES backgrounds who have their first language (L1) as their language of learning and teaching (LOLT) may thus be disadvantaged when compared to their middle class peers in terms of literacy attainment because of the type of language to which the former group receives exposure. However, children from low SES backgrounds who have their second or third language as LOLT may be at an even greater disadvantage. The reason for this is that poverty-situated children with a non-L1 as LOLT receive language input of inferior quality (lacking certain complex language structures) but also of inferior quantity (Huttenlocher, Vasilyeva, Waterfall, Vevea & Hedges, 2007).

### Research question

We report here on an investigation of the comprehension and production of quantifiers by isiXhosa-speaking Grade 1 learners in schools situated in low SES areas who have either isiXhosa or English as LOLT. The following research questions were posed:

- What progress do these learners make in quantifier comprehension and production during their Grade 1 year?
- How, if at all, does the answer to Research Question 1 differ for the English LOLT and the isiXhosa LOLT groups?

### Study rationale

Nippold (2004) concludes that the more that is learned about the nature of later language development (such as investigated in the current study), the relation it has to literacy, and the factors which underlie its growth, the more insight the researcher will gain into the difficulties that children encounter with language in the school context. Such difficulties may manifest not only in children with specific or other language impairment, but also in children who develop typically according to the norms of their community but find themselves in contexts in which the quality and quantity of their linguistic input are not ideal for optimal language and literacy development.

## Quantifiers

### Quantifiers defined

A quantifier is a word or short phrase which indicates the amount or quantity of an object that is referred to by the noun phrase which the quantifier modifies (Southwood & Van Dulm, 2012a). The format of this quantification must allow a distinction between properties, on the one hand, and individuals possessing these properties, on the other (Braine & O’Brien, 1998; Brooks & Sekerina, 2005/2006; O’Brien *et al*. 2003). Quantifiers are functional categories (Radford, 2004) lacking specific descriptive content. As such, they can modify any semantic noun class where grammatical restrictions do not prohibit such modification. Because quantifiers modify noun phrases and determine the quantificational properties of noun expressions, quantifiers generally act as a type of determiner (Radford, 2001).

Beghelli and Stowell (1997) state that quantifiers can be categorised in several manners. Radford (2004), for instance, categorises quantifiers as universal, existential, or partitive. Universal quantifiers are defined as ‘free-choice’ quantifiers such as *all/both* (Radford, 2009). In contrast, the meaning of existential quantifiers relates to the existence of some entity. For instance, *some* in *There is some coffee in the pot* refers to coffee that actually exists (unlike *any* in *Is there any coffee left?*, which questions the existence of coffee) (Radford, 2009). Partitive quantifiers quantify part of the members of a given set, as *some* in *Some children like broccoli* or *any* in *Do any children like broccoli?* (Radford, 2009). Roeper (2007) distinguishes between collective and distributive quantifiers, where *all* is collective and *every* distributive. Quantifiers can also be either prenominal (occurring before the noun, as in *Do you have any books?*) or pronominal (standing on their own, as in *Do you have any*?). Both prenominal and pronominal quantifiers can occur in either the subject or the object position of a sentence. Beghelli and Stowell (1997) base their categorisation of quantifiers on the syntax of quantifier scope. Scope pertains to the referential dependencies between the quantifier phrase and the clause in which it occurs (Beghelli, 1993). For instance, the meaning of *every* is ‘all possible’, but *every* (1) has scope over the subject of the sentence in *Every boy sees the dog*, (2) has scope over the object in *The boy sees every dog*, and (3) assumes scopal ambiguity in *Every boy sees a dog*, where either every boy sees the same dog or every boy sees a different dog.

### The development of quantifiers in child language

Quantifier development has been studied intensively, and the relevant theoretical developments and empirical studies are rich and varied. In Table 1, we provide a summary of the available findings on the age of acquisition of specific quantifiers. Note that there is information available on quantifier acquisition in one of the languages relevant to the current study (English), but not on the other (isiXhosa). Afrikaans data are provided alongside English data in Table 1 to show that there are differences in age of acquisition between these two typologically similar languages. It could thus be assumed that there will also be differences between English and isiXhosa, which are typologically dissimilar. Below the table, we provide a brief exposition of how the quantifiers in the table are formed in English and in isiXhosa.

**TABLE 1:** Age of acquisition of quantifiers per meaning and scope.

Quantifier	Non-South African literature		Receptive and Expressive Language Activities (Southwood & Van Dulm, 2012b)	
	
	Age of acquisition	Source(s)	Age of acquisition for English	Age of acquisition for Afrikaans
**Comprehension of the meaning of the quantifier**				
*all*	4	Brooks and Braine (1996); Roeper (2007).	4	4
*any*	4	Huang and Crain (2014); O’Leary (1994); Song (2003); Thornton (1995).	4	4
*no/none*	4	Hanlon (1987).	4	n/a
*more*	3;3-5	Halberda, Taing and Lidz (2008); Odic, Pietroski, Hunter, Lidz and Halberda (2012).	n/a	5
*most*	3;7	Papafragou and Schwartz (2006); Stickney (2003).	n/a	5
*many*	around 5	Gathercole (1985); Odic *et al*. (2012).	n/a	5
*every*	after 5	Philip (1995); Roeper (2007); Roeper, Strauss and Pearson (2006).	4	5
*some*	4-6	Foppolo, Guasti and Chierchia (2012); Katsos (2009); Papafragou and Musolino (2003); Roeper and Matthei (1974).	5	8
**Comprehension of the scope of the quantifier**				
*all*	Not indicated in studies as meaning and scope were not differentiated in the available literature, with the exception of Southwood and Van Dulm (2012a)		4	4
*every*			4	5
*some*			4	6
*more*			n/a	after 6
*most*			n/a	after 6
*many*			n/a	after 6
*any*			4	7
*no/none*			n/a	n/a

No literature on the production of these quantifiers could be traced for Afrikaans, apart from Southwood and Van Dulm (2012a), which states that production of quantifiers by Afrikaans-speaking children is mastered only after the age of 9 years. There is also a dearth of literature on child L2 acquisition of quantifiers. Available studies (e.g. Lakshmanan, 1995) focus on theoretical and psycholinguistic phenomena and how these would affect children’s developing L2 grammars, but there are little empirical data on child L2 acquisition of quantifiers.

### How quantifiers present themselves in English and isiXhosa

#### Quantifiers in English

English quantifiers include but are not limited to *every, all, any, many/more/most, no/none*, and *some* and are invariant forms which do not undergo any inflection. They can be prenominal as in *All students are welcome* or pronominal as in *All are welcome* (Radford, 2001), but not all quantifiers can be used both prenominally and pronominally in English; for example, pronominal *every* is grammatical (as in *Every student wants to graduate*) but pronominal *every* not (as in *Every wants to graduate) (Radford, 2001). Some English prenominal quantifiers can occur as one-word units (when occurring with nouns, as in *Many* *people are relieved*) or as part of constructions (when occurring with nouns or pronouns, as in *Many of the people/them are relieved*). Quantifiers can have generic reference, and then the zero article is used (as in *All men are handsome*) or specific reference, and then the definite article with or without *of* is used (as in *All* (*of*) *the men are handsome*).

#### Quantifiers in isiXhosa

The isiXhosa quantifiers -*nke* ‘every/all’, -*nye* ‘some’, -*phi* ‘any’, -*ninzi* ‘many’ and -*ngaphezulu* ‘more’ have variant forms where different inflectional processes derive the quantifier, in contrast to *inkoliso* ‘most’ and *a*- ‘no/none’ which are invariant and where only a single morpheme acts as the quantifier. The quantifiers -*nke* ‘every’ and *a*- ‘none’ illustrate this difference between variance and invariance: The quantifier stem -*nke* combines with the quantifier root -*o*- and the relevant subject agreement (according to the noun class of the noun that the quantifier modifies), as in (1). However, the isiXhosa equivalent of *every* can also be expressed by the quantifier form *elowo* or by a combination of the adjective stem *nga*- and -*nye* (in the case of a distributive reading), as in (2):

1. *Wonke umntwana uyadlala*

Wo-nke um-ntwana u-ya-dlal-a

Subject morpheme. Second person.

Quantifier root-every Noun class 1-child Subject morpheme 1-ASPECT-play-PRESENT. Final vowel

‘Every child is playing’ (= all children – collective)

2. *Umntwana ngomnye uyadlala*

Um-ntwana nga-um-nye u-ya-dlal-a

Noun class 1-child Preposition-Noun class 1-one (every) Subject morpheme 1-ASPECT-play-PRESENT. Final vowel

‘Every child is playing’ (= every single one – distributive)

Whereas negation in English can occur by using a quantificational modifier with the noun (as in *no child*), such negation in isiXhosa occurs on the verb. The isiXhosa equivalent of the English quantifiers *no* and *none* is expressed by adding the negative prefix *a*- to the verb, as in (3):

3. *Andizifuna iiapile*

A-ndi-zi-fun-a ii-aplile

NEGATIVE-Subject morpheme.First person-Object morpheme 10-want-PRESENT. Final vowel Noun Class 10-apples‘ I want no apples’/‘I do not want apples’

## Research design and method

### Design

The current study forms part of a larger project on later-developing language skills in young school-going Afrikaans-, English-, and isiXhosa-speaking children (see Nel, 2014). In this study, the comprehension and production of quantifiers at the beginning and end of the Grade 1 year were assessed amongst isiXhosa-speaking children from two schools, one with English as LOLT and the other with isiXhosa as LOLT. Data were collected in the LOLT of the learners. The study was empirical and had a longitudinal and cross-sectional design: The nature of Research Question 1 (which asks what development in the comprehension and production of quantifiers takes place between the start and the end of Grade 1) lends itself to a longitudinal design, by examining changes that occur over the course of a school year. In the cross-sectional part of this study, participants were grouped according to LOLT to see if and how the level of mastery of quantifiers differs between these two groups (to answer Research Question 2). Data were collected with an action research approach. The latter involves a cyclic process in which researchers follow a series of steps that include planning, observing, and evaluating the effects of a specific action which is to be researched (Gray, 2004).

### Participating schools and participants

The study had to be conducted in two similar schools attended by L1 isiXhosa-speaking learners, one with English as LOLT and the other with isiXhosa. The selection criteria for the schools were as follows:

- situated in communities with low SES
- a National Quintile of 3 or lower (i.e. non-fee-paying schools)
- the same geographical classification (either both rural or both urban).

We approached several schools about participating in the study. The only two that consented to participate also met the selection criteria. The English LOLT school is parallel medium, with an Afrikaans and an English stream. It is situated in a rural area 10 km from the nearest town centre. Its 923 learners live on the surrounding farms and in various nearby informal settlements. The school has one educator per 34 learners and a National Quintile of 1. The participants from this school (14 male; 16 female) had a mean age of 6.6 years (range 6.0 years – 7.6 years) at the first point of data collection.

Of the 30 English LOLT participants, 21 were exposed to only isiXhosa in their homes, one to isiXhosa and isiZulu, seven to isiXhosa and English, and one to isiXhosa, English, and Afrikaans. Thirteen of the English LOLT participants were born in Stellenbosch and never moved away. The region in which they had received input and in which language acquisition had taken place has thus been stable and homogenous in comparison to nine other participants. (No data were available on the remaining eight participants.) Of those nine participants, four were born in Cape Town and their parents later moved to Stellenbosch, whereas three were born in Johannesburg and another two in the Eastern Cape. The ninth participant grew up in the Western Cape, but it is not specified where. The place of birth plays an important role, because it means that the regional languages and language varieties to which the participants may have been exposed are heterogeneous, and this might affect the characteristics of their language. Unfortunately, there was no indication of the age at which the children’s families moved to Stellenbosch. In terms of the exposure to other languages outside of the home, the English LOLT participants formed a heterogeneous group: 18 attended a playschool or pre-Grade 1 educational facility (21 facilities in total) during their early childhood years; no data on this were available for the remaining 12 participants. For seven participants, isiXhosa was the language of instruction during their preschool years, for two English, for one Afrikaans, for another one a combination of English and isiXhosa, for three a combination of Afrikaans, English, and isiXhosa and for one a combination of Afrikaans and English. No data on language of instruction was available for the remaining three of those 18 participants who attended a preschool facility.

The isiXhosa LOLT school is in a township adjacent to the industrial area of the same town, 3.4 km from the town centre. It is one of two primary schools in the township. It has 1494 learners and one educator per 40 learners. Its National Quintile ranking is 1. At the first point of data collection, the mean age of the participants from this school (15 male; 16 female) was 6.8 years (range 6.0 – 8.11 years).

Of the 31 isiXhosa LOLT participants, only one was exposed to another language in addition to isiXhosa at home, namely to Afrikaans. Sixteen of the isiXhosa LOLT participants were born in Stellenbosch and never moved away. Four of the other 15 were born in Cape Town and their parents later moved to Stellenbosch whereas one was born in Tygerberg Hospital (which means that the parents lived somewhere in the Western Cape Province at that time) and one in the Eastern Cape. Another participant was born in the Western Cape but the specific place was not indicated. The children who were not born in Stellenbosch will have language profiles that look dissimilar to those children who have stayed in the Stellenbosch area their whole lives. It was however not indicated at which time in the children’s lives their families moved to Stellenbosch. For 8 of the participants, it was not indicated where they were born. Approximately a quarter of the children in the isiXhosa LOLT group had not attended a playschool during early childhood. Twenty-three of the 31 participants attended a total of 17 different institutions, with some overlap between those institutions attended by the English and isiXhosa LOLT groups. The range of language input and the exposure to languages in the isiXhosa group in these pre-Gr 1 educational settings was wide: nine participants received input in isiXhosa only, another nine in English and isiXhosa, and one in English only.

### Data collection material

The Quantifiers booklet of the Receptive and Expressive Activities for Language Therapy (REALt; Southwood & Van Dulm, 2012b) served as data collection instrument. The REALt material was designed for use as language intervention material with children from four to nine years who exhibit a language delay or have a language disorder, but can be used as an informal language assessment instrument (Southwood & Van Dulm, 2012a). Its authors also state that it is suitable for L2 speakers of English as well as for children from poverty-situated communities which can profit from directed language stimulation to aid with language development (Southwood & Van Dulm, 2012a). Since its publication, the English version was translated to isiXhosa by the REALt authors and these two versions were then used for data collection in this study.

The quantifier section of the REALt includes six different quantifiers: *all* (isiXhosa: -*nke*), *any* (-*phi*), *every* (-*nke/nga*-+-*nye*), *many/more/most* (-*ninzi*/-*ngaphezulu/inkoliso*), *no/none* (*a*-) and *some* (-*nye*). Four quantifiers (*all*/-*nke, every*/-*nke*/-*nga*-+-*nye, no/none/a*-, and *some*/-*nye*) have one set of a comprehension items and another set of production items, whereas *any*/-*phi* and *many/more/most*/-*ninzi*/-*ngaphezulu/inkoliso* have comprehension items only. Each of these sets, apart from those of *no/none/a*-, has two subsets, one targeting quantifier meaning (five items each for comprehension and three for production) and another targeting quantifier scope (again five and three items for comprehension and production, respectively). The meaning items comprise stimulus questions based on a picture, requiring either *yes/no* or other one-word responses. The scope items comprise a picture selection task in which learners are presented with a verbal stimulus to which they respond by choosing that one out of three pictures that matches the stimulus.

The REALt assesses production of only *every*/-*nke/nga*-+-*nye, all*/-*nke, some*/-*nye,* and *no/none/a*- as appropriate picture material could not be generated for *any/* -*phi* and *many/more/most*/-*ninzi*/-*ngaphezulu/inkoliso* (Southwood & Van Dulm, 2012a). *Every*/-*nke/nga*-+-*nye, all*/-*nke,* and *some*/-*nye* each has six production items, of which the first three assess the meaning and the last three the scope of these quantifiers. The scope items differ in format from the meaning items, in that there are two parts to each scope item, namely an (a)-part which aims to elicit a response containing a quantifier with a specific scope, and a (b)-part which aims to elicit the same quantifier but with contrasting scope. The quantifier *no/none/a*- has only five production items which appear in a question answering task.

### Data collection and analysis

Data were collected from each learner individually in his or her LOLT by four fieldworkers. The Quantifiers booklet of the REALt was administered to 61 isiXhosa-speaking Grade 1 learners (30 with English and 31 with isiXhosa as LOLT) at the beginning of the first term of 2013 (henceforth also ‘February’). These learners were reassessed in the same manner in the fourth term of 2013 (henceforth also ‘November’). Responses were documented on paper scoresheets. Where spontaneous revisions occurred, the participant’s last response was recorded. If a targeted response was given, the fieldworker made encouraging remarks such as ‘Good job!’ or ‘Well done’. In the case of an incorrect or non-target response, the fieldworker followed up with a somewhat more elaborate version of the initial stimulus. Correct responses to follow-up were scored as correct. No further opportunities for correct response were provided after the one follow-up. Responses were then transferred from the scoresheets to custom designed excel sheets.

The following were calculated separately for comprehension and production of each quantifier, with a score of 90% or more taken as an indication of mastery:

1. the percentage of responses correct for each subtype per learner
2. the average of these percentages for each subtype per LOLT group
3. the average percentage of all comprehension subtypes and of all production subtypes collectively per LOLT group.

The Wilcoxon matched pairs test was used to compare the data collected at the beginning and at the end of the year for each LOLT group separately, with *p* < 0.05 as significance level. The direction of significance (whether scores were better in February or in November) was determined by means of inspecting Box and Whiskers plots.

The data for the English LOLT group and the isiXhosa LOLT group were also compared to each other, in order to answer Research Question 2. This was done by means of the Mann–Whitney U test (with continuity correction), where English data collected in February were compared to isiXhosa data collected then, and English data collected in November to isiXhosa data collected then. The direction of significance was again derived from inspection of Box and Whisker plots. The programme used for statistical analyses was Statistica 12.

#### Ethical considerations

Ethical clearance for the study was granted by the Research Ethics Committee (Humanities) of Stellenbosch University (Protocol number GL010812). Permission to conduct the study was obtained from the principals and relevant teachers at the two study schools. Informed consent was obtained from participants’ parents or legal guardians by distributing information letters and consent forms to them via the school. The letters explained in plain language and in the LOLT of the particular school the general purpose of the study, the procedures to be followed during data collection and reporting of the findings, and the voluntary nature of participation. Participants gave written assent after the fieldworkers had explained to them in isiXhosa what research is and what tasks they would be expected to perform. Participants and their parents or guardians were also informed that participation could be terminated at any point without them having to provide a reason for doing so or being penalised in any manner. Confidentiality and anonymity were assured during all stages of the research process, amongst others by assigning participant codes instead of using participant names.

### Reliability and validity

The following measures were taken in order to increase the reliability and validity of the obtained data:

1. The REALt was administered exactly as stipulated in the manual (Southwood & Van Dulm, 2012a).
2. Data were collected by a small number of fieldworkers (two per school), with no changes in school assignment between the beginning and the end of the year.
3. All fieldworkers received the same intensive training on, amongst others, the research protocol and task administration and scoring.
4. During data collection, responses were recorded and scored on the scoresheets in real time (directly after being given). Each non-target response was written down verbatim. At the end of each data collection day, the first author verified the accuracy of the scoring. Any ambiguity or inconsistency was cleared up with the fieldworker before the next data collection day.
5. Accuracy of data transfer to the excel sheet was checked.

## Results

### Quantifier comprehension

#### English LOLT group

Participants who had English as LOLT did not obtain a 90% score on the comprehension tasks (meaning and scope) for any of the six quantifiers tested, neither in February nor in November, apart from on the comprehension task for meaning of *no/none.* In February, the average comprehension score for meaning of *no/none* was 76%, but in November it was 97% (Table 2). Despite the general lack of mastery, scores for comprehension of meaning increased statistically significantly from February to November, with the exception of *some* which remained the same (Table 2). Regarding scores for comprehension of scope, only *every* and *many/more/most* showed a statistically significant increase (Table 2).

**TABLE 2:** English LOLT and isiXhosa LOLT quantifier comprehension scores: Descriptive statistics.

Variable (see text for isiXhosa equivalents)	English LOLT						isiXhosa LOLT					
	
	Mean %		Standard deviation		*p*-value for difference between beginning and end of year	% variance between mean scores	Mean %		Standard deviation		*p*-value for difference between beginning and end of year	% variance between mean scores
			
	February	November	February	November			February	November	February	November		
Meaning: *every*	47	67	0.308	0.304	**0.002**	+20	90	88	0.199	0.191	0.700	−2
Scope: *every*	41	67	0.203	0.254	**< 0.000**	+26	91	88	0.162	0.169	0.514	−3
Meaning: *all*	47	79	0.330	0.265	**< 0.000**	+32	89	95	0.218	0.146	0.221	+6
Scope: *all*	49	57	0.261	0.256	0.070	+8	85	72	0.251	0.211	0.054	−13
Meaning: *some*	55	55	0.233	0.221	0.770	0	81	81	0.121	0.197	0.274	0
Scope: *some*	40	49	0.235	0.227	0.142	+9	81	76	0.216	0.250	0.550	−5
Meaning: *any*	61	71	0.285	0.256	**0.044**	+10	94	95	0.140	0.152	0.600	+1
Scope: *any*	32	45	0.238	0.227	0.051	+13	81	73	0.294	0.251	0.339	−8
Meaning: *many/ more/most*	43	66	0.282	0.183	**0.002**	+23	88	86	0.219	0.183	0.918	−2
Scope: *many/ more/most*	37	57	0.221	0.194	**< 0.000**	+20	85	80	0.237	0.208	0.357	−5
Meaning: *no/ none*	76	97	0.318	0.109	**0.005**	+21	96	100	0.184	0.000	0.180	+4
Comprehension: TOTAL Score	47	64	0.175	0.127	**0.000**	+17	87	85	0.156	0.108	0.294	−2

LOLT, language of learning and teaching.

#### isiXhosa LOLT group

In the isiXhosa LOLT group, comprehension scores for the meaning subsets of *a*- ‘no/none’ and -*phi* ‘any’ indicated mastery in February, with a further increase in November. The meaning subset of -*nke* ‘all’ was fully acquired at the end of Grade 1: The average percentage increased from 89% in February to 95% in November. The score for the meaning subset of -*nke/nga*-+-*nye* ‘every’ was 90% in February but decreased slightly to 88% in November. This quantifier was thus almost fully acquired during Grade 1. The remaining quantifiers in the meaning subset had high average percentages and were almost fully acquired in November (Table 2). The scores of the scope subset decreased slightly from the beginning to the end of the year, but this decrease was not statistically significant. The scores for the scope subset are not as high as those for the meaning subset, and comprehension of the scope of the assessed quantifiers were not mastered by the end of G1.

### Quantifier production

#### English LOLT group

In the English LOLT group, quantifier production errors mainly included quantifier omission (such as *the boys are kicking the balls* instead of *all the boys are kicking the balls*), the use of definite articles instead of a quantifier (such as *they picked the flowers* instead of *they picked some flowers*), circumlocutions with negation (such as *I don’t see red* instead of *There are no red balloons*) and irrelevant picture description. No quantifiers were fully acquired by November as none of the average percentages were above 90, but scores for both the meaning and the scope subsets of all the quantifiers showed an increase from February to November. Not all increases were statistically significant though (Table 3). For the meaning subset of *every, all* and *some*, there was a statistically significant increase but not for *no/none*. For the scope subset, only the quantifier *all* showed a significant increase. Both in February and November, meaning scores were higher than scope scores (Table 3).

**TABLE 3:** English LOLT and isiXhosa LOLT quantifier production scores: Descriptive statistics.

Variable (see text for isiXhosa equivalents)	English LOLT						isiXhosa LOLT					
	
	Mean %		Standard deviation		*p*-value for difference between beginning and end of year	% variance between mean scores	Mean %		Standard deviation		*p*-value for difference between beginning and end of year	% variance between mean scores
			
	February	November	February	November			February	November	February	November		
Meaning: *every*	36	55	0.334	0.292	**0.014**	+19	95	95	0.136	0.155	1.000	0
Scope: *every*	29	42	0.318	0.360	0.105	+13	96	95	0.105	0.117	0.814	−1
Meaning: *all*	56	88	0.441	0.239	**0.001**	+32	100	100	0.000	0.000	n/a	0
Scope: *all*	38	56	0.372	0.275	**0.009**	+18	98	99	0.050	0.030	0.361	+1
Meaning: *some*	31	63	0.391	0.354	**0.001**	+32	99	100	0.060	0.000	n/a	+1
Scope: *some*	34	45	0.408	0.364	0.134	+11	100	95	0.000	0.115	**0.028**	−5
Meaning: *no/ none*	23	36	0.324	0.380	0.121	+13	99	95	0.036	0.152	0.178	−4
Production: TOTAL Score	35	53	0.307	0.237	**0.005**	+18	98	97	0.041	0.052	0.372	−1

LOLT, language of learning and teaching.

#### isiXhosa LOLT group

Quantifier production errors made by the isiXhosa LOLT participants mainly comprised quantifier omission, circumlocutions with negation (*umtwana omnye akanaye unonkala* ‘the other child doesn’t have a crab’ instead of *oononkala bahamba ezinyaweni zabanye babantwana* ‘the crabs are running over some of the children’s feet’), and irrelevant picture description or other irrelevant responses (such as *bahamba ngenyawo* ‘walking barefoot’ instead of *abanye oononkala baqabela ngaphaya kweenyawo zabantwana* ‘some crabs are running over the children’s feet’). The isiXhosa LOLT participants had production scores for meaning and for scope of 90% or higher in February and in November for all quantifiers. Meaning and scope scores were almost similar in February and again in November. The increases or decreases from February to November show no specific pattern, and none of the differences between February and November were significant apart from the score for the scope subset of -*nye* ‘some’ which showed a significant decrease. Despite this decrease, the November score was still above 90% (Table 3).

### Comparison of English LOLT and isiXhosa LOLT scores

**FIGURE 1:**
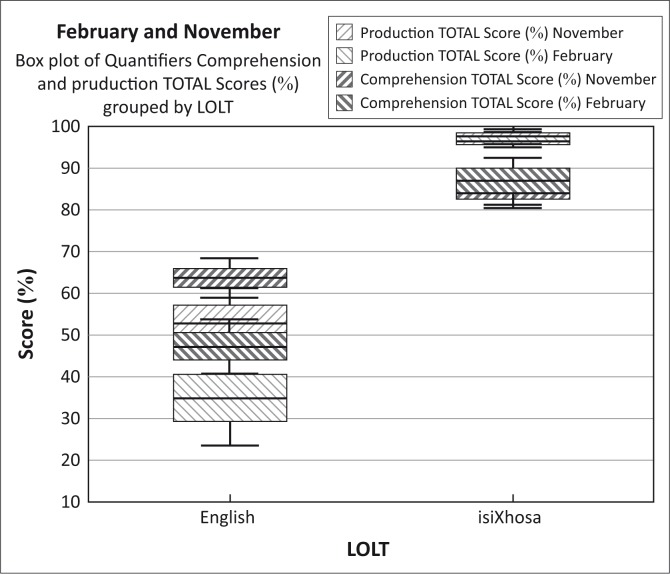
The comprehension and production total scores of all quantifier types (%) per language of learning and teaching.

A comparison between the English and the isiXhosa LOLT learners shows a clear distinction between these two groups in terms of both their comprehension and production skills, with the learners who have their mother tongue as LOLT consistently obtaining higher scores (80% – 95%) than those who have their L2 as LOLT (50% – 60%). For each quantifier, subset, and set, there was a statistically significant difference between the two LOLT groups, apart from comprehension of the meaning of *no/none/a*- (*p* = 0.507) and production of the meaning of *all*/-*nke* (*p* = 0.075). In these latter two cases, the scores of the isiXhosa LOLT group were however still higher than those of the English LOLT group; see Figure 1 which is a Box and Whiskers plot of the total scores for comprehension and production for the two LOLT groups. Table 4 is a summary of the ages of mastery of comprehension and production of the quantifiers assessed in the current study.

**TABLE 4:** Age of acquisition of comprehension and production of quantifiers based on data from the current study

Quantifier	Comprehension		Production	
	
	English	isiXhosa	English	isiXhosa
	**Comprehension of meaning**		**Production of meaning**	
*all* / -*nke*	After 6;9–8;2	6;10–9;7	After 6;9–8;2	6;1–8;11
*any* / -*phi*	After 6;9–8;2	6;1–8;11	Did not assess	Did not assess
*every* / -*nke/nga*-+-*nye*	After 6;9–8;2	After 6;10–9;7	After 6;9–8;2	6;1–8;11
*many/more/most* / -*ninzi*/-*ngaphezulu/inkoliso*	After 6;9–8;2	After 6;10–9;7	Did not assess	Did not assess
*no/none /a*-	6;9–8;2	6;1–8;11	After 6;9–8;2	6;1–8;11
*some* / -*nye*	6;9–8;2	After 6;10–9;7	After 6;9–8;2	6;1–8;11
	**Comprehension of scope**		**Production of scope**	
*all* / -*nke*	After 6;9–8;2	After 6;10–9;7	After 6;9–8;2	6;1–8;11
*any* / -*phi*	After 6;9–8;2	After 6;10–9;7	Did not assess	Did not assess
*every* / -*nke/nga*-+-*nye*	After 6;9–8;2	After 6;10–9;7	After 6;9–8;2	6;1–8;11
*many/more/most* / -*ninzi*/-*ngaphezulu/inkoliso*	After 6;9–8;2	After 6;10–9;7	Did not assess	Did not assess
*some* / -*nye*	After 6;9–8;2	After 6;10–9;7	After 6;9–8;2	6;1–8;11

## Discussion

Based on the available literature on the age of acquisition of quantifiers (Table 1), quantifiers seem to be early developing rather than later-developing, with the exception of the quantifier *every*. However, a distinction between meaning and scope is not always made in the relevant literature. From the data obtained in the current study, it is not conclusive whether quantifiers are earlier- or later-developing in isiXhosa L1 speakers: Comprehension of *a*- ‘no/none’ and -*phi* ‘any’ has been mastered by the beginning of Grade 1 (age 6.1 – 8.11) by isiXhosa-speaking children, which could indicate early development. However, comprehension of the remaining quantifiers is only mastered later, pointing to later development. Different quantifiers are thus mastered at different ages. This could be because of differences in the inflectional processes involved in the various isiXhosa quantifiers. Also, the complex processes which a child has to apply in order to interpret a quantified phrase correctly might play a role in why some quantifiers prove to be more difficult for learners than others quantifiers. In this regard, Brooks and Sekerina (2005/2006) state that:

although quantifiers play a very important role in logical reasoning …, their acquisition may be delayed relative to other sorts of lexical items (e.g. nouns and verbs) because their complex patterns of usage often result in interpretive ambiguities. (p. 177)

Despite the fact that the isiXhosa LOLT group had not yet acquired the comprehension of -*nke/nga*-+-*nye* ‘every’, -*nke* ‘all’, -*nye* ‘some’, and -*ninzi*/-*ngaphezulu/inkoliso* ‘many/more/most’ by the end of Grade 1, quantifier production is already fully acquired at beginning of Grade 1. A possible reason why the production scores are higher than the comprehension scores could be that the production items, unlike the comprehension items, do not provide opposer and distracter pictures which could confuse the learners. As stated above, errors in the production data comprised the omission of a quantifier, circumlocutions, irrelevant picture description, and responses completely unrelated to the stimulus, but not substitution of one quantifier with another. The learners thus did not produce untargeted quantifiers as there was no stimulus priming other quantifiers. Based on the results of this study, one can conclude that isiXhosa Grade 1 learners who have isiXhosa as LOLT have sufficient command of quantifiers when they enter Grade 1 to allow them to understand classroom discourse containing this syntactic category.

Turning to the isiXhosa-speaking learners with their L2 as LOLT: None of these learners achieved a percentage correct score of 90% or more for quantifier comprehension (not even at the end of their Grade 1 year), and their scores were significantly lower than those of their peers with isiXhosa as LOLT. Despite the fact that the English LOLT learners had various ages of first exposure to English, ranging from no exposure prior to entering Grade 1 to exposure from birth or preschool entrance, none of them had mastered quantifier comprehension at school entry, and all required more than one year’s exposure to English classroom discourse to master quantifier comprehension. Note however that there was a significant increase in the comprehension and production scores of English quantifiers from the beginning to the end of Grade 1. The increase in the English LOLT group’s scores was statistically significant. Thus, despite the fact that these quantifiers are not yet fully acquired by the end of Grade 1, significant development takes place between the beginning and the end of Grade 1. In this regard, Jordaan (2011) found that L2 learners who are integrated with L1 language learners catch up with these L1 language learners by Grade 3. Jordaan (2011) also states that although those L2 learners who are not integrated with L1 peers do make significant progress by the end of Grade 3 on most of the language skills that she assessed, they do not reach the same level as those integrated into a L1 learning context. The learners in the English LOLT group of the current study find themselves in the latter situation: They are L2 learners of English in a class consisting of mainly L2 learners of English. They have received and still receive limited English input, because their main (and, in many cases, only) source of English input is their teachers who are not English L1 speakers. Although these learners are supposed to be taught through medium of English only (as per the language policy of the school), their teachers are ‘forced’ to code switch in class (between English and isiXhosa) because of the learners’ low level of English comprehension and production at the beginning and, to a large extent, still at the end of Grade 1 (personal communication with teachers). The school’s language policy is thus not always followed in practice, as it is not practical (or even possible) in all teaching contexts. This group of isiXhosa-speaking learners is at a disadvantage compared to those in a classroom with isiXhosa as LOLT, because in the absence of code switched classroom discourse, they have limited ability to access complex English sentences such as those containing quantifiers. By contrast, isiXhosa-speaking learners receiving their tuition in their L1 will be adequately prepared to understand quantifiers in the narratives and numerical literacy tasks they encounter in the Grade 1 classroom.

## Conclusion

The study investigated the quantifier comprehension and production of children from low SES backgrounds who consequently might not receive adequate linguistic input at home for the acquisition of complex language constructions. When comparing the scores of these low SES learners with those obtained by the higher SES learners assessed by Southwood and Van Dulm (2012a), it is clear that the age of acquisition is later for the low SES isiXhosa L1 speakers who receive their schooling in their L1 and even more so for those who receive their schooling in English. Children who are situated in low SES circumstances are generally impoverished in terms of the language input they receive, and the language development norms applicable to middle or high SES communities cannot necessarily be applied to low SES communities. This study rendered limited normative data on the acquisition of quantifier constructions by child speakers of isiXhosa and contributed to the still small pool of normative data on the language acquisition of older children. In summary in this regard, there seems to be a general acquisition order for the meaning subset: -*phi* ‘any’ and *a*- ‘no/none’ are acquired earlier than -*nke* ‘all’, and -*ninzi*/-*ngaphezulu/inkoliso* ‘many/more/most’, -*nye* ‘some’, and *nke/nga*-+-*nye* ‘every’ are acquired thereafter. The findings of this study support the notion that child language acquisition has not been completed by the age of five years, thus challenging certain assumptions about language acquisition and developmental norms. Despite the fact that children can generally construct most sentence types and decode complex semantic relationships in their L1 upon school entry, their language continues to develop in significant manners during at least their first years of school.

The study had several limitations. These included a limited number study schools in only one geographical location; group generalisations instead of careful consideration of possible individual variation; combining the results of the two genders instead of searching for possible gender-related differences; considering grade instead of chronological age; and focusing on learner responses only instead of imbedding the study in classroom observations so as to ascertain the potential influence of teacher talk and pedagogical styles on learners’ linguistic knowledge. Despite these limitations of this study, the findings have a practical implication: Children with no or very little prior knowledge of their LOLT will generally need either, (1) more deliberate exposure to quantifier rich language in their LOLT, or (2) longer exposure to general classroom discourse in their LOLT before quantifiers can be expected to be mastered sufficiently to allow access to quantifier-related curriculum content. Without such exposure, children from low SES backgrounds for whom there is a mismatch between their L1 and LOLT are unlikely to function optimally in the Grade 1 classroom.
